# Evaluation of *KIR3DL1*/*KIR3DS1* allelic polymorphisms in Kenyan children with endemic Burkitt lymphoma

**DOI:** 10.1371/journal.pone.0275046

**Published:** 2023-08-30

**Authors:** Beatrice M. Muriuki, Catherine S. Forconi, Erastus K. Kirwa, Titus K. Maina, Bonface O. Ariera, Jeffrey A. Bailey, Anita Ghansah, Ann M. Moormann, John M. Ong’echa

**Affiliations:** 1 West African Center for Cell Biology of Infectious Pathogens, College of Basic and Applied Sciences, University of Ghana, Accra, Ghana; 2 Center for Global Health Research, Kenya Medical Research Institute, Kisumu, Kenya; 3 Division of Infectious Diseases and Immunology, Department of Medicine, University of Massachusetts Chan Medical School, Worcester, MA, United States of America; 4 Department of Pathology and Laboratory Medicine, Warren Alpert Medical School, Brown University, Providence, RI, United States of America; 5 Noguchi Memorial Institute for Medical Research, College of Health Sciences, University of Ghana, Legon, Accra, Ghana; Food and Drug Administration, UNITED STATES

## Abstract

Endemic Burkitt lymphoma (eBL) is a fast-growing germinal center B cell lymphoma, affecting 5–10 per 100,000 children annually, in the equatorial belt of Africa. We hypothesize that co-infections with *Plasmodium falciparum* (*Pf*) malaria and Epstein-Barr virus (EBV) impair host natural killer (NK) and T cell responses to tumor cells, and thus increase the risk of eBL pathogenesis. NK cell education is partially controlled by killer immunoglobulin-like receptors and variable expression of KIR3DL1 has been associated with other malignancies. Here, we investigated whether KIR3D-mediated mechanisms contribute to eBL, by testing for an association of *KIR3DL1*/*KIR3DS1* genotypes with the disease in 108 eBL patients and 99 healthy Kenyan children. *KIR3DL1* allelic typing and EBV loads were assessed by PCR. We inferred previously observed phenotypes from the genotypes. The frequencies of *KIR3DL1*/*KIR3DL1* and *KIR3DL1*/*KIR3DS1* did not differ significantly between cases and controls. Additionally, none of the study participants was homozygous for *KIR3DS1* alleles. EBV loads did not differ by the *KIR3DL1* genotypes nor were they different between eBL survivors and non-survivors. Our results suggest that eBL pathogenesis may not simply involve variations in *KIR3DL1* and *KIR3DS1* genotypes. However, considering the complexity of the *KIR3DL1* locus, this study could not exclude a role for copy number variation in eBL pathogenesis.

## Introduction

Endemic Burkitt lymphoma (eBL) is a fast-growing germinal center B cell lymphoma, affecting pediatric patients within Papua New Guinea and tropical Africa [1]. It is a multifactorial disease, where risk factors such as genetic, environmental, and childhood infections cooperate to cause pathogenesis [2]. It is well documented that most Burkitt lymphoma (BL) cases over-express the *c-myc* oncogene, due to chromosomal translocation, t(8:14) [3], which results in uncontrolled cell growth. In Kenya, eBL prevalence is high in the western region where *Plasmodium falciparum* (*Pf*) transmission occurs throughout the year [4]. Furthermore, about 90% of eBL cases are infected with Epstein-Barr virus (EBV); [5] a chronic infection usually acquired by the age of 2 years in Africa [6]. Since eBL affects children aged 0–14 years old, these observations have led to the speculation that the early age of co-infections with EBV and repeated malaria increases the risk of eBL tumorigenesis [7, 8]. Malaria induces immune down-regulation that influences immune surveillance over EBV by natural killer (NK) and T cells [9]. This immunomodulation results in the accumulation of a pool of EBV-infected B cells [10], viral reactivation, and higher viral loads contributing to the etiology of eBL [7, 11]. NK cells are lymphocytes involved in antiviral and anti-tumor immunity [12]. Their education and licensing involve the acquisition of inhibitory and activating killer immunoglobulin-like receptors (KIRs) [13], which are also expressed by some T cells [14]. Variations in gene content, alleles and copy numbers of KIR genes influence individuals susceptibility to diseases and treatment outcomes [15, 16]. Interaction of KIRs with human leukocyte antigen class I (HLA-I) ligands on the target cells enables licensed NK cells to recognize and tolerate self or to kill target cells lacking “self” human leukocyte antigen class I [13, 17, 18]. A balance of inhibitory and activating signals is crucial for NK cell education [19]. Therefore interruption of inhibitory signal, through the interaction of mature NK cells with HLA-deficient viral-infected or tumor cells may activate NK cells [20]. Consequently, enhanced NK cell activation is associated with pathogenesis in some virus-associated diseases, probably due to non-specific inflammatory responses [21–24]. Furthermore, blocking KIR3DL1 receptor enhances NK cell cytotoxicity of target cells [25]; explaining the possible role of KIR3DL1 in NK cells self-inhibition. The licensing concept explains how this inhibitory KIR may influence NK cells’ antiviral and antitumor potency, through altered NK cell cytolytic activities [25]. KIR3DL1 is a highly polymorphic KIR whose locus encodes inhibitory (KIR3DL1) and activating (KIR3DS1) allotypes (IPD-KIR sequence database: http://www.ebi.ac.uk/ipd/kir/) [26]. The *KIR3DL1/3DS1* gene has over 200 alleles [27]; which can be classified into three genotypes *3DL1*/*3DL1*, *3DL1*/*3DS1*, or *3DS1*/*3DS1* [28]. Considering the different patterns of expression and homology, the alleles can be categorized into functionally distinct KIR3DL1*High, KIR3DL1*Low, KIR3DL1*Null, and *KIR3DS1 (3DS1)* genotypes [29, 30]. KIR3DL1 recognizes *HLA*-Bw4 and *HLA*-Bw6 ligands and their interactions are categorized into strong, weak, and non-interacting [31]. Their binding avidity may influence NK cell immune responses. *KIR3DS1* interacts with *HLA-F* ligands [32].

Functionally, variations in *KIR3DL1* alleles have different influences on diseases. For example, *KIR3DL1-High* and HLA-Bw4 *057 are associated with better outcomes in HIV infected individuals [33]. Generally, this receptor-ligand interaction would generate strong NK inhibitory signals. *KIR3DL1-Low* alleles have been implicated in the onset of psoriasis, probably because of enhanced immune responses associated with the disease [34]. In contrast, the same genotype had better outcome in chronic myeloid leukemia, suggesting that decreased inhibitory signal enhanced NK cells cytolytic activities in chronic myeloid leukemia patients [35]. Therefore, given the importance of NK cells in the control of viral infected and tumor cells [36], the functionality of different *KIR3DL1* genotypes may provide insights into eBL pathogenesis. Our previous study evaluated the presence and/or absence of 16 KIR genes and reported that individuals with an increased number of activating KIRs had a high risk of eBL pathogenesis [21]. To further our understanding on the role of KIR polymorphisms in eBL pathogenesis, the current study evaluated the association of *KIR3DL1* alleles with EBV load, eBL susceptibility and survival in the same Kenyan population.

## Materials and methods

### Study participants

This retrospective study analyzed available DNA from 108 eBL patients and 99 healthy children (HC) from Kenya. Sample size was determined logically, based on available DNA. Patients with eBL were enrolled at Jaramogi Oginga Odinga Teaching and Referral Hospital, a regional referral hospital in western Kenya, between 2007 and 2012, and were aged 0–13.5 years old. The eBL diagnosis was performed from fine-needle aspirates as previously described [37]. The eBL patients were treated with a combination of cyclophosphamide, vincristine, methotrexate, prednisone, and Adriamycin (CHOP), which is the standard therapy for eBL [38]. The healthy controls were conveniently sampled from children aged 0–12 years old, with a healthy medical history and no known history of cancer. They were living in the same malaria-endemic areas of western Kenya, as the eBL patients between 2005 and 2012.

### Ethical approval

This study was approved by the Scientific and Ethics Review Unit at the Kenya Medical Research Institute and the Institutional Review Board at the University of Massachusetts Chan Medical School, Worcester, USA. Written informed consent was obtained from the parents and/or guardians before enrollment. Assent was sought from children aged 13 years and above as per the local institutional review board guidelines.

### DNA extraction and KIR3DL1 genotyping

Genomic DNA was extracted from blood samples using QIAGEN QIAamp® (Valencia CA, USA). *KIR3DL1* typing was performed by customized nested real-time PCR applying Taqman genotyping assay, using the probes and PCR conditions adapted from a published protocol [39]. We distinguished the alleles which were functionally expressed on the surface of NK cells (including *001, *002, *008, *009, *015, *020, *029, *035, *005, *007, and *053 and *3DS1*) from the non-functional *KIR3DL1* alleles (*004 and *019, which were retained intracellularly) [30]. Only *KIR3DL1* alleles with a frequency greater than 1% in the African population were investigated, those with a frequency less than 1% were ignored [40]. Taqman probes (Applied Biosystems, Streetsville, ON, Canada) were used to differentiate between common *KIR3DL1* genotypes. The allele expression levels were inferred from the genotypes as previously described [39]. *KIR3DS1* and *KIR3DL1* were considered alleles of the same locus. *KIR3DL1* locus with high sequence similarities was analyzed by a nested real-time PCR strategy. Amplification was performed on an Eppendorf flexlid nexus gradient Mastercycler as instructed by the manufacturer. The PCR amplification conditions were: 90s at 94°C, 30s at 94°C (30 cycles), 30s at 56°C—plate read, 30s at 72°C. The PCR reactions were optimized as previously described [39]. The product of amplification was verified by gel electrophoresis and then diluted one in 10^6^, to provide a template for custom Taqman genotyping assays. All assays were performed according to the manufacturer’s instructions for Taqman Genotyping Master Mix (Applied Biosystems).

### Definition for KIR3DL1 allelic polymorphisms

KIR3DL1*High alleles are densely expressed on the cell surface and strongly inhibit NK cell-mediated lysis of tumor cells. KIR3DL1*Low are lowly expressed on the surface of NK cells and generate weak inhibition signals to NK-cell mediated cytolysis. KIR3DL1*Null alleles are not expressed on the cell surface, but have intracellular retention, with minimal inhibitory signaling. *KIR3DS1* generates activating signals. The KIR3DL1 phenotypes were inferred from the observed genotypes as previously reported [39]. Individuals carrying at least one high allele but no low allele were considered as KIR3DL1*High carriers, while individuals carrying at least one low allele were considered KIR3DL1*Low carriers. If an individual had one null allele and no copy of either a low or high allele, they were considered a *Null phenotype [29].

### Determination of EBV load

EBV load was determined by qPCR as previously described [41]. Briefly, DNA was extracted from 200μl whole blood using the Qiagen DNA easy kit (Qiagen) following the manufacturer’s instructions and stored at -20°C until use. Standard curve dilutions were made by adding DNA to each tube sequentially. A volume of 2μl of sample and 13μl of master mix (BioRad Laboratories, Hercules, CA Cat. No.170-8860) were added to the bottom of the center of the well to bring the total well volume to 15μl. Amplification was done in a BioRad CFX96 Real-Time System with a C1000 Thermocycler base for the primers and probes. The PCR amplification conditions were: 180s at 95°C, 10s at 95°C, 30s at 63.5°C, 10s at 95°C (39 cycles).

### Statistical methods

This genetic association study applied the STREGA assessment (STrengthening the REporting of Genetic Association Studies) [42]. *KIR3DL1/KIR3DS1* frequencies were calculated by direct counting and expressed as the percentage of the study population having the trait in eBL patients and healthy controls. Fisher’s exact tests were used to test the association between *KIR3DL1* genotypes and the risk of eBL. Comparisons were done using the HC as the reference group. Survival was defined as the interval between the hospital admission and the date of last follow-up or death and was computed using the Kaplan-Meier method. Differences between subgroups were tested by log-rank tests. Kruskal–Wallis test and Mann-Whitney test were used to compare log-transformed EBV load between the genotypes and between eBL survivors and non-survivors respectively. The statistical significance of associations was assessed using odds ratios (OR) with 95% confidence intervals (CI). Statistical analyses were performed in R version 3.6.1 (The R Foundation for Statistical Computing) and Graphpad Prism version 8.0.2 (GraphPad Software, La Jolla, CA). The results are reported using the median and *p-*value. A *p*-value less than or equal to 0.05 was considered statistically significant, while a *p*-value greater than 0.05 was indicated as non-significant (ns).

## Results

### Patients’ characteristics

To assess the association of *KIR3DL1* polymorphisms with eBL, we typed 108 Kenyan patients with eBL and 99 healthy controls. The median age of eBL patients at diagnosis in our study population was 8.2 years, (interquartile range (IQR):6.2–10.3), and 66.7% of eBL patients were males. Controls were healthy volunteers derived from the same community, without a history of childhood cancers. Their median age at the time of enrollment into the study was 6.1 years, (IQR:3.4–8.3), and were 56.1% males. The peak onset of eBL ranges from 5–9 years of age (8, 9). Malaria positivity was 20.0% (18/90) and 39.0% (30/77) in eBL patients and HC, respectively. Among eBL patients, 35.0% (36/103) individuals died, while the rest were alive at the last follow-up. The patients were followed for 2 years post-diagnosis.

### Distribution of *KIR3DL1* alleles in eBL patients and healthy controls

Typing of *KIR3DL1* allowed us to distinguish 3 distinct functional genotypes, with different allele combinations. We observed that none of the genotypes were associated with eBL. Additionally, no specific combination of *KIR3DL1*/S1 alleles conferred protection or risk to eBL (**Table 1**).

**Table 1:** Frequency of *KIR3DL1* alleles with different predicted cell expression levels in eBL patients and healthy Kenyan children.

*KIR3DL1*/S1 Alleles					
Allele 1	Allele 2	eBL	HC	Odds Ratio	*p-* value
		n = 108 (%)	n = 104 (%)	(95% Confidence Interval)	
High genotype					
3DL1*High	3DL1*High	55 (50.93)	53 (53.54)	0.901 (0.521–1.550)	0.781
3DL1*High	3DL1*Null	10 (9.26)	6 (6.06)	1.582 (0.599–3.930)	0.444
3DL1*High	*3DS1*	10 (9.26)	6 (6.06)	1.582 (0.599–4.520)	0.444
Low genotype					
3DL1*High	*3DL1* ^*^ *Low*	26 (24.07)	20 (20.20)	1.252 (0.638–2.399)	0.616
*3DL1* ^*^ *Low*	*3DL1* ^*^ *Low*	2 (1.85)	1 (1.01)	1.849 (0.212–27.040)	1.000
*3DL1* ^*^ *Low*	3DL1*Null	1 (0.93)	2 (2.02)	0.453 (0.031–3.960)	0.607
*3DL1* ^*^ *Low*	*3DS1*	2 (1.85)	5 (5.05)	0.355 (0.070–1.380)	0.262
Null genotype					
3DL1*Null	3DL1*Null	2 (1.85)	2 (2.02)	0.915 (0.141–5.930)	1.000
3DL1*Null	*3DS1*	0	4 (4.04)	0.000 (0.000–0.680)	0.060
*3DS1* genotype					
*3DS1*	*3DS1*	0	0	N/A	0

Abbreviation: n, number of subjects

### Distribution of KIR3DL1 genotypes in eBL patients and healthy controls

All the study participants carried at least one *KIR3DL1* allele (n = 207), hence we did not observe any homozygotes for *KIR3DS1* (*KIR3DS1*/*KIR3DS1*) genotype (i.e. individuals who do not carry any copies of *KIR3DL1*). In addition, there were no individuals negative for both alleles. The frequency of homozygous *KIR3DL1* (*KIR3DL1*/*KIR3DL1*) was 88.89% and 84.85%, while heterozygous *KIR3DS1* (*KIR3DL1*/*KIR3DS1*) had a frequency of 11.11% and 15.15% in eBL patients and HC respectively. The KIR3DL1*High, KIR3DL1*Low, and KIR3DL1*Null genotypes represented 69.44% vs 65.65%, 28.70% vs 28.28%, and 1.85% vs 6.06% in eBL patients and HC respectively (**Table 2**). No *KIR3DL1* genotypes were associated with eBL.

**Table 2:** Analysis of diploid *KIR3DL1* genotypes in eBL patients and healthy Kenyan children.

Diploid *KIR3DL1*	eBL	HC		Odds Ratio	*p-* value
	n = 108 (%)	n = 99 (%)		(95% Confidence Interval)	
*KIR3DL1*/*KIR3DL1*	96 (88.89)		84 (84.85)	1.430 (0.657–3.320)	0.415
*KIR3DL1*/*KIR3DS1*	12 (11.11)		15 (15.15)		
*KIR3DS1*/*KIR3DS1*	0	0			
KIR3DL1 genotype					
KIR3DL1*High	75 (69.44)	65 (65.65)		1.19 (0.668–2.120)	0.656
KIR3DL1*Low	31 (28.70)	28 (28.28)		1.02 (0.549–1.830)	1.000
KIR3DL1*Null	2 (1.85)	6 (6.06)		0.292 (0.059–1.220)	0.156

Abbreviation: n, number of subjects

### KIR3DL1 genotypes and survival of eBL patients

To evaluate whether *KIR3DL1* genotypes were associated with a patient’s survival, we analyzed the *KIR3DL1* genotypes between eBL survivors and the non-survivors **(Table 3)**.

**Table 3:** The percentage of *KIR3DL1* genotypes in eBL survivors compared to non-survivors.

*KIR3DL1 g*enotype	eBL survivors	eBL non-survivors	Odds Ratio	*p-* value
	n = 67 (%)	n = 36 (%)	(95% Confidence Interval)	
KIR3DL1*High	47 (70.15)	25 (69.44)	1.030 (0.432–2.490)	1.000
KIR3DL1*Low	18 (26.87)	11 (30.56)	0.835 (0.337–2.030)	0.819
KIR3DL1*Null	2 (2.99)	0		0.541

Abbreviation: n, number of subjects

**Fig 1:**
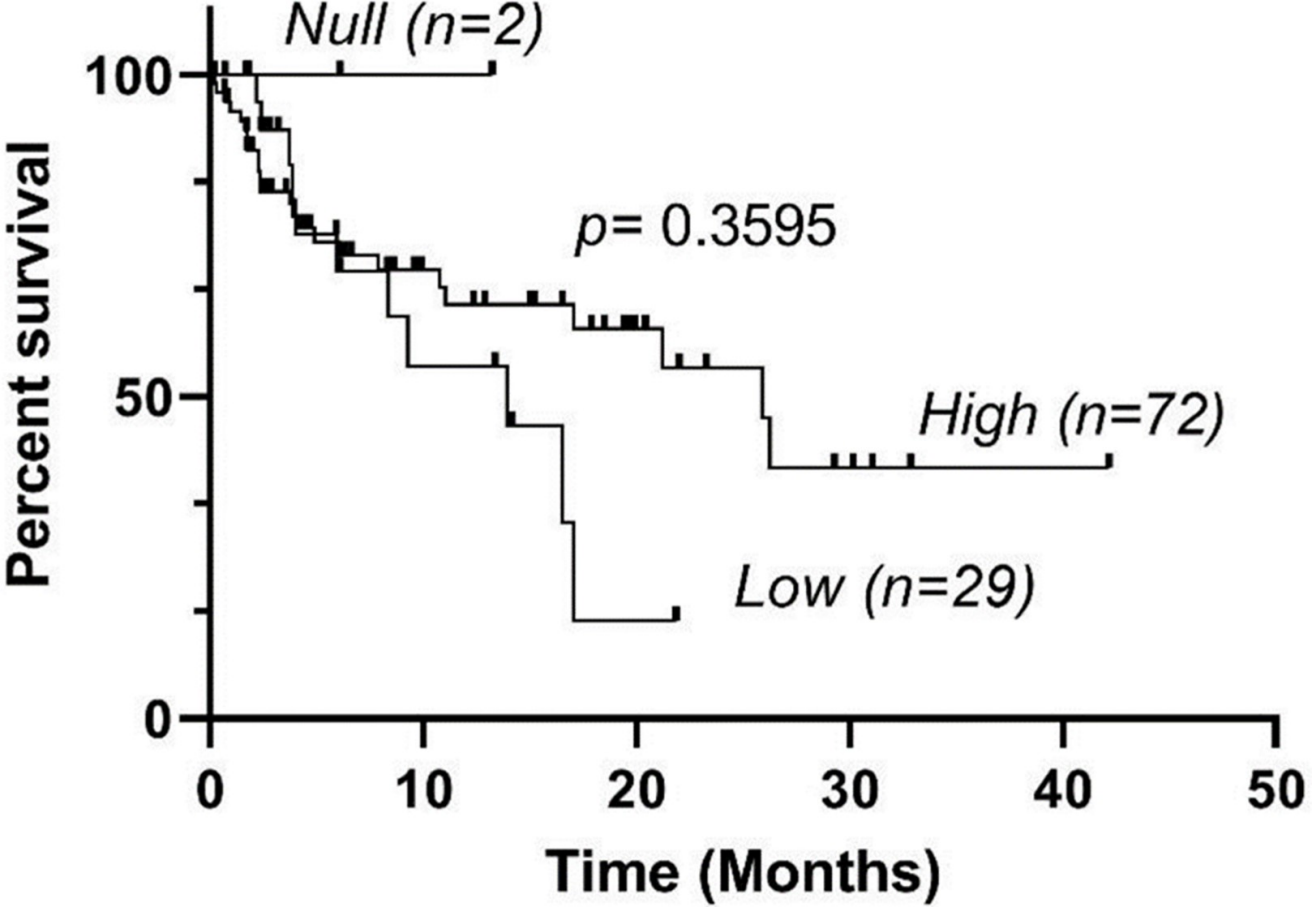
*KIR3DL1* genotypes and overall survival (OS) in patients with eBL. OS of eBL patients encoding KIR3DL1*High, KIR3DL1*Low, and KIR3DL1*Null genotypes were analyzed using the Kaplan-Meier method.

In the study population, 65% (67/103) of eBL patients survived and 35% died (36/103). We did not have 2-year survival data for 5 eBL patients. We observed no significant differences in survival among eBL patients with the *High*, *Low* and *Null KIR3DL1* genotypes (**Fig 1**).

### *KIR3DL1* genotypes and EBV loads in eBL patients and healthy controls

**Fig 2:**
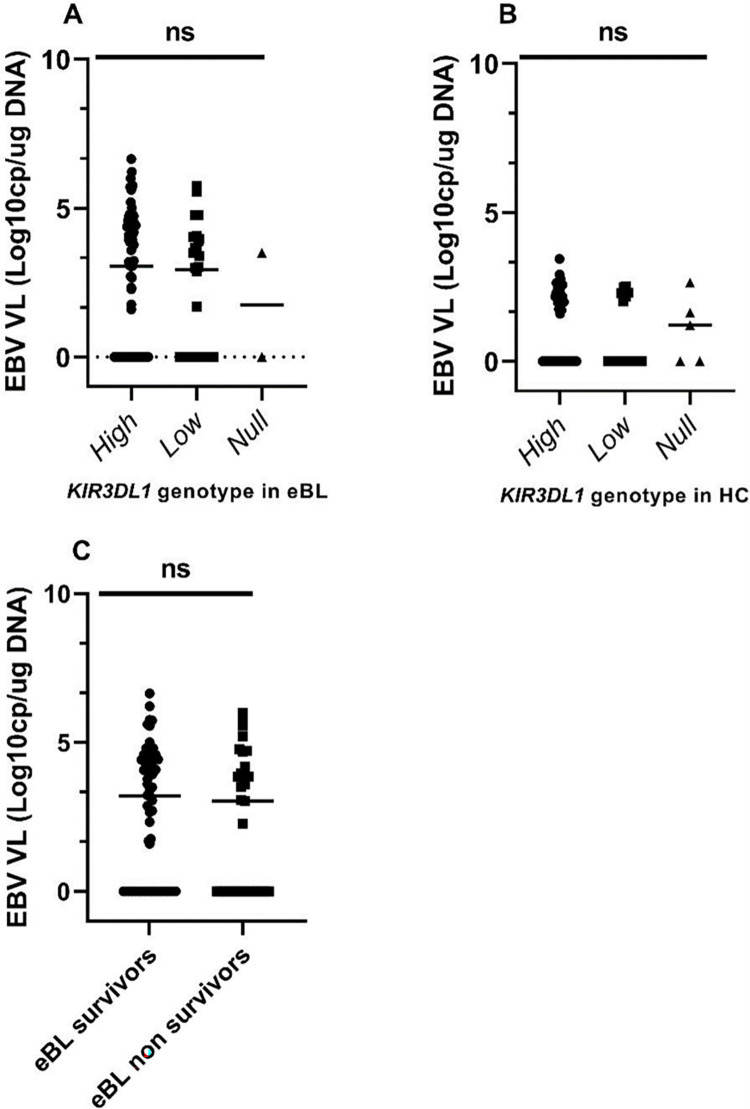
EBV load stratified by *KIR3DL1* genotypes and by survival in eBL patients. Cellular EBV levels were compared for (A) eBL patients (n = 102) and (B) HC (n = 78) after stratification by *KIR3DL1* genotypes. In addition, EBV levels were compared between eBL survivors and non-survivors (C). Analyses were performed by Kruskal–Wallis and Mann-Whitney tests. *p*≤0.05 was considered statistically significant. VL is viral load. Data are median, ns, not significant.

To determine whether EBV loads differed by the *KIR3DL1* genotypes, we analyzed the available viral loads for 102 Kenyan patients with eBL and 78 HC. As expected, children with eBL had significantly higher viremia (median 1099.54 EBV copies/ug of DNA) compared to healthy children (median 0 EBV copies/ug of DNA) (*p-value* <0.0001) (9). However, the median EBV load was not different between *KIR3DL1* genotypes (**Fig 2A and 2B**). As previously reported, high EBV load was associated with an increased risk of eBL (OR = 3.072; 95% CI 1.108–5.6814; *p* = 0.006), [43] but not independently associated with the patient’s survival (**Fig 2C)**.

## Discussion

NK cells destroy tumor cells lacking HLA ligands with varying efficiency [44], depending on receptor-ligand binding affinity, avidity, and cell surface density [45]. *KIR3DL1* alleles have variable inhibitory strength upon ligation to *HLA-Bw4* and *HLA-Bw6* ligands [31]. We previously demonstrated that NK cells from eBL patients have increased density of inhibitory KIR3DL1, and have limited ability to kill target K562 tumor cells *in vitro* relative to healthy controls [9]. The current study investigated the possible association of *KIR3DL1* genotypes with pathogenesis and survival in eBL patients. Overall, the Kenyan study population had the *KIR3DL1* genotype, and no individual had the *KIR3DS1* genotype. Unlike non-African populations where diversifying selection has maintained the *KIR3DL1-High*, *KIR3DL1-Low*, and *KIR3DS1* genotypes at equivalence, directional selection favors the *KIR3DL1-High* genotype among the African population [40] Consequently, *KIR3DL1-Low* is uncommon while *KIR3DS1* is rare among Africans [40, 46]. Consistent with these findings, *KIR3DL1-High* alleles had a higher frequency than *KIR3DL1-Low* alleles in the Kenyan study population. Previous studies have demonstrated that the density of KIR3DL1 impacts NK cell functions where KIR3DL1-High interacts with its HLA-ligand to generate a strong inhibitory signal [31]. Since NK cells are sensitive to cells with decreased expression of HLA-I, the strength of interaction of KIR3DL1 and HLA-B subtypes determines the degree of NK inhibition and hence their anti-tumor activities [40]. Consequently, the stronger the inhibition that prevents an NK cell from attacking healthy cells, the stronger its response toward unhealthy cells [18, 47]. Natural selection would therefore select for NK cells with stronger responses to infections [40]. Exposure of Africans to acute and chronic infections [48], might favor the selection of individuals with potent NK cells that are sensitive to decreased expression of HLA-I in the African population [40]. Considering this hypothesis, we expected KIR3DL1*High to provide a selective advantage through enhanced clearance of tumor cells, thus protecting individuals from eBL pathogenesis. We would therefore expect eBL patients to be less associated with the KIR3DL1*High genotype. On the contrary, the frequencies of *KIR3DL1 g*enotypes were not different between eBL patients and healthy controls in our study population.

*KIR3DL1* alleles have been reported to differentially influence outcomes within the context of other cancers. While poor overall survival was observed in weakly interacting and non-interacting alleles in metastatic colorectal cancer patients on chemotherapy [49], the same phenotypes were associated with favorable outcomes in patients with neuroblastoma, treated with anti-GD2 monoclonal antibody [31]. Furthermore, strongly interacting *KIR3DL1* genotypes were associated with poor overall survival [31, 45]. Individuals with KIR3DL1*High genotypes have an increased percentage of NK cells abundantly expressing KIR3DL1 phenotype [50]. The strong interaction of this genotype with its *HLA-Bw4* ligands would strongly inhibit NK cells under normal circumstances [29, 39], hence weakening their immune responses. In some instances, the lack of NK inhibition may be beneficial for successful NK responses [51], as evidenced by enhanced survival in patients with lymphoma receiving rituzimab treatment [52]. Furthermore, blockade of *KIR3DL1-high* and *Bw4* interaction has been shown to restore the effector functions *in vitro* [31]. In contrast, our results did not demonstrate significant differences in overall survival between eBL patients with different *KIR3DL1* genotypes. However, since KIR3DL1 is a complex locus with sequence and copy number variations [53], future micro-array experiments will be needed to evaluate duplications, deletions and rearrangements which results in varying numbers of copies of *KIR3DL1* and *KIR3DS1* in each chromosome and their impact on eBL etiology.

Highly educated NK cells are important effectors in antiviral, anti-tumor, and antimalarial immune responses. Infections with *Pf* alter the NK cells subsets and the KIR/HLA repertoire, subsequently affecting NK cell responses to malaria [48]. In the African population, chronic malaria infections down-regulate NK and T cell responses, resulting in elevated EBV loads [9, 11]. Consequently, evolutionary pressure from malaria pathogens might have selected KIR/HLA combinations that protect against severe malaria but which increase the risk of other diseases [48]. In the context of other infectious diseases, the role of *KIR3DL1* and their *HLA* ligands is controversial, with some studies associating them with protection from HIV and AIDs [20, 33] and others with disease severity in COVID-2019 [54]. Interestingly, malaria-exposed children and eBL patients express the KIR3DL1*High phenotype [9], a proposed mechanism by which malaria subverts NK-cell mediated immune responses [9]. In contrast, our analysis, while requiring replication, did not associate the predicted KIR3DL1*High phenotype with increased risk of eBL in the same study population. Considering EBV infections, the association between EBV and outcome in lymphomas is controversial, with some studies reporting an association [55] and others no association [56]. In our study, EBV loads were not different between the *KIR3DL1* genotypes in the study population.

Our study has some limitations. First, although KIR/HLA combinations influence individual’s susceptibility to diseases and may exert selective pressure in populations [57], due to limited genomic material, our study focused only on analysis of *KIR3L1/3DS1* alleles using the available DNA. We therefore did not perform HLA typing experiments for this study population. Convenience sampling of the HC led them to being younger when compared with eBL patients. However, since *KIR3DL1* alleles do not differ by age, we believe that convenient sampling would not bias the findings. In addition, our conclusions were limited by the small number of study participants with the *KIR3DL1 High*, *Low*, and *Null* genotypes.

## Conclusion

Our findings, while requiring replication, add to the existing body of literature on KIRs and eBL pathogenesis. While our previous study associated activating KIRs with eBL pathogenesis [21], the current study extends these observations to exclude the association of inhibitory *KIR3DL1* alleles in eBL pathogenesis. Since the frequencies of *KIR3DL1* and *KIR3DS1* alleles are not significantly different in eBL patients compared to healthy Kenyan controls, they do not appear to independently increase the risk of eBL. In addition, the alleles do not influence EBV viral loads, suggesting the possibility of other mechanisms inhibiting NK cell antiviral and anti-tumor activities in eBL patients. Considering that maximum NK cell education is dependent on a high density of receptors and ligands and their binding strength [45] and that the *KIR3DL1* alleles impact the strength of interaction with its *HLA*-*Bw4* and *HLA-Bw6* ligands, further studies with larger sample sizes are required to explore the influence of varying strengths of receptor-ligand interactions on NK cell anti-tumor and anti-viral activities in eBL patients.

## Supporting information

S1 File Contain the datasets evaluated in this study. (XLS)
